# Large trials of psychosocial interventions: examples from individual placement and support

**DOI:** 10.3389/ijph.2026.1609143

**Published:** 2026-06-09

**Authors:** Justin D. Metcalfe, Kim T. Mueser, Robert E. Drake

**Affiliations:** 1 Westat, Bethesda, MD, United States; 2 Boston University, Boston, MA, United States; 3 Columbia University Vagelos College of Physicians and Surgeons, New York, NY, United States

**Keywords:** individual placement and support, pilots, psychosocial intervention, randomized controlled trials, validity

## Abstract

**Objectives:**

Large demonstration projects for health interventions often use randomized controlled trials (RCTs) to test the effectiveness of interventions implemented at larger scales, serving as crucial contributors to policy and funding decisions. Such trials are subject to limitations common to all RCTs, but their size and importance magnify the costs of failure to satisfy the assumptions for valid causal inference and generalizability. We examine common reasons for such threats to validity.

**Methods:**

We examined large (N > 1,000) IPS RCTs for aspects of design and execution that undermine the validity of their results.

**Results:**

We identified three large IPS RCTs and identified threats to validity associated with treatment adherence and attrition.

**Conclusion:**

Large trials should rely on pilot studies to ensure that difficulties with recruitment, implementation of and participation in interventions, and follow-up measurement do not compromise study validity; intervention fidelity and participation should be measured to permit evaluation of study success. Funders should require and support the use of pilot studies and other prior research to justify the introduction of an intervention to a population and anticipate potential threats to validity.

## Introduction

The use of large trials to comprehensively evaluate psychosocial interventions is an established practice that can determine those interventions’ effectiveness and identify the practical considerations associated with delivering the interventions at scale. The high costs associated with such projects magnify researchers’ responsibility to design and implement successful and informative studies, as invalid results can be misleading and undermine adoption of an otherwise promising intervention. These projects often utilize randomized controlled trial (RCT) designs, which provide the strongest inferential basis for drawing causal conclusions about the relative effectiveness of the intervention on outcomes compared to one or more other treatments. Large studies of mental health interventions typically report smaller effect sizes than small studies [1]; while this can be due to the greater accuracy inherent in the larger sample sizes and wider range of settings, methodological problems can also reduce the apparent effect of interventions [2]. The history of Individual Placement and Support illustrates the opportunities and pitfalls that large and small RCTs face in facilitating the development, validation, and adoption of a widely implemented, evidence-based intervention.

The basic conditions for valid causal inference with an intent-to-treat analyses of RCTs—often referred to as internal validity--include a sample large enough to detect the chosen effect size, random treatment arm assignment, participation in assigned treatment, and minimal loss to follow-up [3]. External validity, a concept that includes generalizability, suitability, and transferability, describes the extent to which a study’s causal results hold over a range of people and settings beyond the those observed in the study [4]. Standards for evaluation of external validity have not been established and are therefore less likely to be systematically assessed for a given RCT [5]. External validity is typically assumed from the outset in the form of enrollment criteria and the selection of intervention implementation settings, which are usually selected to represent the desired population and their manner of service receipt. These assumptions face threats from recruitment errors yielding a sample that does not reflect the intended study population, overly restrictive inclusion criteria, and implementation of interventions in a manner not reflective of how they would be delivered in actual practice [6, 7]. Without careful preparation, these conditions can prove elusive.

Individual Placement and Support (IPS) is a form of supported employment that improves rates of competitive employment among those with serious mental illness (SMI). People with SMI experience substantial functional impairments associated with a diagnosed mental illness. Since publication of the first IPS RCT in 1996, researchers have established the effectiveness of IPS in over 30 RCTs and provided ongoing support to increasing numbers of real-world IPS programs and clients in more than 20 countries [8], including more than 1000 IPS programs in the United States [8–12]. Most of these RCTs have used samples of less than 200, and nearly all took place in real-world settings using typical employment specialists and participants with documented mental disabilities who wanted to pursue competitive employment. This is typical for psychosocial research, as the interventions are complex and must be embedded in the staff, clients, and local environment of the host organization.

Before conducting the first IPS RCT, researchers established the feasibility of IPS for people with serious mental illness [13]. This provided the necessary *a priori* understanding, usually acquired in the form of pilot studies or an equivalent research history, that the program would be both acceptable to the target population (e.g., participants would choose to participate and would remain in the program) and appropriate (e.g., address their needs and goals), and that any obstacles to its implementation would be identified [14]. Foreknowledge of feasibility and potential intervention effect can help investigators anticipate threats to validity when either scaling up an intervention or applying it to a new population (Table 1) [15, 16]. The developers of IPS followed the preferences of people with SMI for competitive employment and studied IPS implementation in a series of pilot studies involving conversions of traditional rehabilitation programs to supported employment programs [17–24]. These studies, relying on methods already developed for recruitment of people with mental illness to supported employment programs [25], drew samples of those with SMI receiving mental health services who did not have jobs but wanted to work and were of working age [13, 26, 27]. Investigators did not provide incentives to encourage participation in IPS, and clients not interested in employment received other supports. IPS researchers developed a thorough working knowledge of this population (adults with SMI who wanted to work) and the implementation context (community mental health programs).

**TABLE 1:** Basic requirements for randomized controlled trial to maintain internal and external validity and specific aspects of study design that pilot studies inform^a^.

Requirement for validity	Relevant pilot domain^b^				
​	Intervention feasibility	Recruitment	Quality assessment	Instruments	Measurement procedures
Study design	​	​	​	​	​
- Clear hypotheses that address questions of relevance to stakeholders	✓	​	​	​	​
- Clinical equipoise (scientific and provider)	✓	​	​	​	​
Population & sampling	​	​	​	​	​
- Clear inclusion/exclusion criteria for participants to ensure generalizability of intent-to-treat findings to population of interest	✓	✓	​	​	​
- Adequate power (sample size) to test hypotheses	​	✓	✓	​	✓
- Randomized treatment arm assignment	​	​	​	​	​
- Effective & consistent recruiting strategy	✓	✓	✓	​	​
Adherence	​	​	​	​	​
- High fidelity implementation of interventions (threats to which include staff turnover, inadequate training, competing treatments/interventions)	✓	✓	✓	​	​
- Active participation of enrollees in assigned treatment (adherence)	✓	✓	✓	​	​
Measurement & attrition	​	​	​	​	​
- Valid measures (blinded interviews, where possible)	​	​	✓	✓	✓
- Minimal loss to follow-up	✓	✓	✓	​	✓
- Intent-to-treat analysis inclusion of all randomized participants in statistical analyses of treatment effects	✓	✓	✓	✓	✓

^a^ This table applies specifically to the employment trials described in this manuscript; standardized criteria for conduct and reporting of randomized controlled trials are available elsewhere [70, 71].

^b^ Pilot Domain Details.

*- Intervention Feasibility* as it applies to proposed population and setting (includes implementation challenges, suitability to population, potential adaptations, willingness of population to participate).

*- Recruitment* (includes rates of recruitment, practical and effective incentives, receptivity to model, referral and outreach methods).

*- Ongoing quality assessment* of recruitment, intervention, and follow-up.

*- Instruments* (including baseline characteristics of participants, outcome measures, process measures).

*- Follow-up measurement procedures* (in-person or telephone self-report interviews and observational data; data collection intervals/timelines, protocols, and systems).

From the earliest RCTs, the screening process included at least two informational sessions prior to consent. Researchers used those interviews to educate prospective recruits about the study (including IPS) and the expectations of them, answer questions, and confirm that recruits understood the study, were still interested in work, and were willing to participate in both the IPS program and the research assessments. Studies measuring IPS participation reported rates in assigned treatment above 90% [13, 27, 28] and minimal attrition from research. In the largest such study that enrolled adults with SMI and reported IPS participation rates, the multi-national EQOLIZE study (N = 213) [29], 87% of those randomized to IPS participated. Meta-analyses of IPS RCTs have reported that those participating in IPS were from 1.63 (95% CI 1.46, 1.82) (11) to 2.40 (95% CI 1.99, 2.90) times more likely to find employment than those in control conditions [30].

Due to the effectiveness of IPS across multiple RCTs and the absence of empirical support for other vocational interventions, clinical researchers have extended IPS to new populations, including people with autism [31–34], posttraumatic stress disorder [35, 36], substance use disorders [37, 38], common mental disorders such as anxiety and depression [39], chronic pain [40], and spinal cord injury [41]. These have typically been pilots and smaller studies that provide critical information regarding population responses to planned recruiting efforts, implementation feasibility, proposed instruments, obstacles to participant study completion, and the potential benefits of the intervention [42, 43]. Pilot findings can help researchers design a recruitment process that addresses the concerns and lifestyles of potential participants and minimizes dropout and non-participation by ensuring that enrollees are eligible and understand their responsibilities [25]; identify adaptations necessary for the intervention to maximize participation and fidelity; and plan how to maintain contact with recruits and incentivize their continued participation [44, 45].

The history of IPS research can serve as a case study in the development and implementation of an evidence-based practice. After a series of small RCTs comparing IPS to usual service controls, a series of large RCTs (N > 1,000) reported unexpectedly small effects. The purpose of this review is to examine threats to validity and their root causes in large IPS RCTs.

## Methods

In this article, we examine threats to validity in large trials of IPS that are generally applicable across psychosocial intervention research. We used existing systematic reviews and a Google Search to identify large (N>=1,000) RCTs with IPS-based interventions compared with control groups receiving usual services and found three studies. We examined final reports and peer-reviewed articles to evaluate threats to valid causal effect estimates of treatment assignment (i.e, internal validity) and how errors in study design and execution can undermine external validity.

## Results

### Three large IPS RCTs

We identified three large, multi-site IPS evaluation projects that included samples of more than 1,000 participants. The first study enrolled a large sample from a population that was very similar to prior IPS studies. In contrast, each of the latter two studies drew samples from populations—for example, recently denied disability applicants—with which IPS researchers had little to no familiarity.

The Mental Health Treatment Study (MHTS), the first large demonstration project featuring IPS, enrolled current Social Security Disability Insurance (SSDI) beneficiaries with SMI (N = 2,055) across 23 sites with established, high-fidelity IPS programs [46]. It benefited both from prior IPS research and site-level experience providing IPS. Over a one-year enrollment period, the MHTS randomized recruits to participate in either IPS (with the addition of a nurse care coordinator) or usual services and followed each for 2 years with quarterly interviews. It used established scientific recruitment methods [25], including two informational sessions prior to enrollment [47]. Those assigned to participate in IPS were 19% more likely to find competitive employment (52% versus 33%) [46].

The Breaking Barriers San Diego (BBSD) study evaluated IPS effectiveness for low-income, unemployed adults or adults who wanted better jobs. Enrollees had a range of self-reported disability types and severities and were drawn from a multi-ethnic, low-income population. The most common disabilities included depression (48%), other psychological disorder (38%), substance use (34%), musculoskeletal injury or other connective disorder (21%), development/learning problems (18%), and heart condition, blood pressure, or other circulatory system disorder (13%). The San Diego Workforce Partnership established BBSD to provide IPS services from January 2016 through June 2018 at four local job centers. Participants were residents of San Diego County receiving services from one of three referral agencies: California’s Temporary Assistance for Needy Families, the California Department of Rehabilitation, or San Diego County Behavioral Health Services [48, 49]. The evaluation enrolled 1,061 subjects served over a 22-month period, randomized them to one of two arms comparing IPS to the usual local services, and followed them for an average of 15 months. IPS was implemented only during the study period and achieved “fair” to “good” fidelity. ITT analysis based on sample not lost to follow-up indicated that 74% of those randomized to the IPS-based treatment arm and 71% of those randomized to receive usual services found employment—a non-significant difference.

The Supported Employment Demonstration (SED) recruited people whose initial applications for either Supplemental Security Income (SSI) or Social Security Disability Insurance (SSDI), based on a claim of mental illness, had been denied (N = 2,944) [50]. The goal was to determine whether provision of IPS to this population could reduce disability and eventual disability awards by improving employment, income, and health. Researchers conducted the study at 30 experienced IPS programs, randomized recruits to a usual-services control arm, consisting of a printed guide to available resources, and Basic- and Full-Service IPS-based treatment arms, and followed them for 3 years [51]. Those randomized to either IPS-based treatment arm received case management services and help with work-related expenses; Full-Service IPS was augmented with a nurse care coordinator who delivered Medication Management Services. As in the BBSD, unemployment was not an eligibility requirement. Over 3 years of follow-up, and based on 60% of the recruited sample, many of whom were only sporadically measured, the SED reported that 74% of those randomized to the IPS-based treatment arms and 64% of those randomized to receive usual services found competitive employment. This was an unexpectedly small effect.

### Threats to the validity of large IPS studies

Table 2 summarizes threats to validity observed in each large IPS RCT.

**TABLE 2:** Fulfillment of requirements for internal & external validity in large randomized controlled trials of Individual Placement and Support (United States, 2008–2022) (✓ = requirement fullfilled; 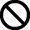 = requirement not fulfilled).

Requirement for validity	Large IPS Study		
​	Mental Health Treatment Study [46]	Breaking Barriers San Diego [48]	Supported Employment Demonstration [50]
Study design			
- Clear hypotheses that address questions of relevance to stakeholders	✓	✓	✓
- Clinical equipoise (scientific and provider)	✓	✓	✓
Population & sampling			
- Clear inclusion/exclusion criteria for participants to ensure generalizability of intent-to-treat findings to population of interest	✓	✓	✓
- Adequate power (sample size) to test hypotheses	✓	✓	✓
- Randomized treatment arm assignment	✓	✓	✓
- Effective & consistent recruiting strategy	✓	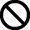	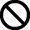
- External validity: *a priori* understanding of population	✓	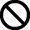	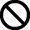
Adherence			
- High fidelity implementation of interventions (threats to which include staff turnover, inadequate training, competing treatments/interventions)	✓	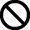	Incomplete measure
- Active participation of enrollees in assigned treatment (adherence)	Unmeasured	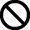	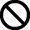
- External validity: Analysis of participation patterns	​	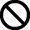	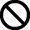
Measurement & attrition			
- Valid measures (blinded interviews, where possible)	✓	✓	✓
- Minimal loss to follow-up	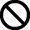	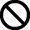	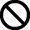
- Intent-to-treat analysis inclusion of all randomized participants in statistical analyses of treatment effects	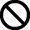	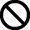	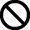
- External validity: Effects of attrition on generalizability	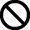	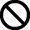	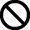

#### Populations and sampling

The MHTS enrolled a sample of people with SMI who had diagnoses of either schizophrenia (32%) or a major mood disorder (68%) who were unemployed at study entry [46]. However, in excluding SMI and unemployment from their eligibility requirements and not approaching participants through mental health centers, the BBSD and SED enrolled samples from populations that differed fundamentally from the original target population of IPS. BBSD enrollees had low rates of SMI and did not usually have ongoing relationships with mental health providers; researchers identified them through their participation in Breaking Barriers employment services. Self-reported baseline assessments indicated that many participants claimed disability due to a mental health disorder: 49% due to depression, 38% due to some other psychological disorder, and 34% due to substance use disorder. The BBSD did not report on the number of enrollees employed at baseline, but 42% had been employed in the past year [48]. Of the SED sample, 19% were already employed at baseline. SED recruits, drawn from lists of recently denied applicants provided by the Social Security Administration, had a broad array of physical and mental health conditions, including an average of 3.5 severe general medical conditions and 91% with a diagnosed mental illness [52]. Unexpectedly, many SED enrollees did not self-identify as having a mental illness and also refused mental health services despite having filed applications for disability to which Social Security Administration reviewers assigned a claim of mental illness [53]. The population for each study was poorly described prior to study design, and investigators had little to no data regarding study feasibility for either population.

#### Recruiting

Despite this lack of precedent, the designs of the BBSD and SED included less rigorous recruiting methods than prior IPS studies, including the MHTS. Researchers had no evidence to suggest that, for example, informing people about the study and enrolling them in the same meeting would be sufficient to enroll a useful sample. The MHTS [47] relied on rigorous scientific recruitment methods [25], including two informational sessions prior to enrollment to confirm understanding of the study procedures and desire to work, that resulted in much higher rates of both participation in assigned treatment and response to data collection efforts than observed in the latter studies. In contrast, the BBSD did not utilize informational sessions prior to enrollment; instead, informed consent followed a single meeting with research staff to determine eligibility. Researchers also noted inconsistent application of eligibility requirements during the recruitment period and skepticism from staff at recruiting partners regarding the potential benefits of participation [48]. The SED offered potential enrollees a range of additional benefits, including help with gaining insurance, and payments for housing, transportation, clothing, dental care, and training, none of which were present in prior IPS studies. Then, after a *single* informational meeting, recruiters screened potential participants for competency and acquired informed consent [54]. Each study enrolled a high percentage of participants who did not engage in either their assigned treatment or the data collection procedures to which they had agreed.

#### Treatment adherence and fidelity

The BBSD and SED each reported poor adherence to treatment assignment. Adherence relies on two primary components, the evaluation of which require careful, ongoing measurement throughout follow-up. First, the assigned intervention must be available to enrollees and administered with fidelity to that intervention’s model. Second, the study enrollees must participate in the intervention. Failure on either point will 1) reduce the observed effect of the intervention because those randomized to it are less likely to receive it and 2) harm internal and external validity by introducing unmeasured confounding into the causal effect of participation in treatment and raising questions about who, in the target population, the intervention would impact. Ineffectively implemented interventions and enrollees who do not participate in services also prevent productive exploration of organizational barriers, such as staff attitudes, and a range of usability concerns in real-world settings [55, 56]. Comprehensive monitoring requires a combination of ongoing qualitative interviews of potential recruits, participants, and study staff, and longitudinal, quantitative measures of intervention fidelity and individual participants’ intervention-related activities. Such monitoring also contributes to a potential secondary goal of any large, pragmatic trial: evaluation of the intervention’s sustainability in the chosen context and suitability to the target population.

IPS programs in BBDS were implemented only during the study and at locations without any experience implementing the intervention. These sites also lacked the capacity to provide mental health services and were therefore unable to integrate employment and mental health services, violating a basic principle of IPS. IPS achieved only “fair” to “good” (never “excellent”) fidelity scores. Researchers were also unable to assess either duration or quality of individual IPS services received because participation measures were unable to discriminate between attempts to contact and actual contact with participants. Measurement of participation relied on a single interview conducted at the end of the trial (15 months after baseline) in which self-reported employment and IPS service participation were assessed. Only two-thirds of those randomized to the intervention group who were interviewed reported participating in IPS services.

In the first two years of the SED, IPS fidelity rose from means of “fair” to “good” before the COVID-19 pandemic halted periodic fidelity evaluations. Early in the study, IPS teams reported unexpectedly low rates of participation in IPS services. Many enrollees made no or minimal effort to follow through with clear expectations for their participation in the interventions; were difficult or impossible to contact; enrolled in the study to obtain its benefits without participating in the research; were avoiding work while appealing disability denials; and otherwise demonstrated behaviors clearly contradictory to their stated intentions of wanting to work [53, 57–60]. The original study design did not include thorough adherence monitoring. During the enrollment period, SED researchers developed the IPS Participation Measure [57], using it to assess and improve participation. After 2 years of follow-up, 20% of those followed with the new measure from enrollment (N = 857) had not participated in IPS, and only 50% had received the recommended 6 consecutive months of the service. In contrast, problems with participation in assigned treatment were not noted in the MHTS. Unless explicitly stated, uptake of and retention in an intervention are not measures of an intervention’s value, but rather are necessary conditions for conducting an internally valid evaluation of the intervention. Researchers did not analyze predictors of participation in the complete samples of any of these studies or otherwise attempt to evaluate external validity.

There are statistical tools available to researchers at study completion that permit estimate of the effect of participation in treatment but which cannot restore validity to a study with poor adherence. These include per-protocol (PP) and as-treated (AT) analyses [61] and causal methods such as instrumental variables and complier adjusted causal effect estimation [62, 63]. These methods rely on measured participation to compare study participants based on differing definitions of treatment adherence. While useful, each is subject to the measured and unmeasured confounding associated with analyses of non-randomized subsets of enrolled samples. The interpretation of each method’s results can vary based on adherence patterns, must be carefully contextualized to avoid overstating the strength of the result, and are best use by researchers to maximize the value of flawed data.

#### Attrition

The difficulties that the BBSD and SED experienced with adherence foreshadowed high rates of study attrition. Of the initial BBSD sample (N = 1,061), 38% were lost to follow-up [48]. Between 25% and 35% of SED enrollees did not complete each quarterly interview, and researchers did not conduct ITT analyses; they instead conducted final analyses based on 60% of the enrolled sample [50]. BBSD researchers found that 74% of those randomized to the IPS-based treatment arms and 71% of those randomized to receive usual services found employment—a non-significant difference. However, subsequent to completion of the BBSD, researchers obtained administrative data from the National Directory of New Hires containing records of employment for the entire sample [49]. They found 68% and 61% first year employment rates for the IPS and control groups, respectively—a statistically significant, if small, effect.

SED researchers attempted to reduce the impact of attrition on effect estimates, in final analyses, with statistical tools. They first discarded the data for the 40% of the sample lacking sufficient survey responses to describe outcomes during follow-up. Then, they created adjusted weights to account for attrition within each study arm. Finally, they used stepwise regression to compare outcomes between treatment arms. Given critical but unmeasured confounding (such as motivation) and the documented shortcomings of stepwise variable selection techniques [64], this combination of tools almost certainly yielded a biased estimate of IPS treatment effectiveness [65]. Low rates of participation would have added to this bias, and SED researchers did not incorporate adherence into effect estimates [50]. Over 3 years of follow-up, and based on 60% of the recruited sample, many of whom were only sporadically measured, the SED reported that 74% of those randomized to the IPS-based treatment arms and 64% of those randomized to receive usual services found competitive employment. This represents a significant difference of 10%—as in the BBSD, an unexpectedly small effect. No attempts were made to assess the impact of attrition on generalizability.

## Discussion

Two of three identified large RCTs of IPS suffered substantial threats to validity when they experienced low rates of treatment adherence and study retention. These flaws indicate poor planning. BBSD and SED researchers were unfamiliar with IPS in the proposed context and poorly informed regarding the targeted populations' attitudes toward employment and employment services, mental illness and mental health services, and ongoing participation in a research project. As a result, the small effect sizes that these studies reported cannot be taken at face value. That those results have not been published in peer-reviewed journals and have only been uncritically presented in government and agency reports distorts the scientific record and undermines the adoption of a potentially beneficial intervention.

Researchers should carefully consider how they would like a study to generalize [66, 67] and be explicit about whether their ITT analysis is intended to represent a per-protocol effect [2]. The study intervention’s integration into services should reflect its implementation in the real world to maximize generalizability, and both intervention fidelity and enrollee participation should be measured to confirm study assumptions and enable detailed analyses of the intervention and its engagement by participants. Each of these large studies assumed that ITT analyses would yield valid estimates of the effects of IPS participation: each attempted to enroll samples that would participate in job services; each funded the IPS services necessary to provide services to all of those participating in IPS; none of the studies included provisions for measuring participation or conducting adherence-based analyses of treatment effects. High rates of attrition only compounded the problems related to poor treatment adherence.

We recommend that investigators pause studies experiencing difficulties with adherence and attrition that lack immediate and obvious solutions. Investigators can study records, formulate potential responses, and consult with funders regarding the wisdom of continuing the trial. Researchers should not succumb to the temptation to draw on available information to understand shortcomings and optimize the enrollment or engagement process while the study is underway. Recruitment, intervention implementation, and data collection plans are extremely difficult to change midstream, threaten study validity, and cannot compensate for an absence of pilot data. Such remediation efforts are akin to attempting deferred maintenance from the driver’s seat while motoring down the highway. Researchers should also not continue the study under the assumption that problems with participation in assigned treatment and missing data can be addressed at the analytic stage. The statistical tools remaining at study completion to compensate for low rates of participation or follow-up are sufficient only to quantify the extent of the study’s failure to meet its own objectives, contextualize any published findings, and inform future researchers making a similar attempt. In other words, the failed study becomes the precedent it lacked at its commencement.

Differences between the investigators who conduct small versus large RCTs may offer a partial explanation for the flagrant methodological shortcomings of some large RCTs despite a copious literature [68, 69] describing psychosocial trials and threats to their validity. Small studies tend to be initiated by investigators with a primary interest and expertise in psychosocial treatment outcome research involving specific interventions and procedures. In contrast, funders and contractors with the capacity to conduct large scale evaluations may lack familiarity with the intervention and target population, and overestimate feasibility for the proposed study. Those designing large RCTs may also assume that such trials have sufficient statistical power to overwhelm problems with participation or attrition. They may also view participation in the interventions (or lack thereof) as one indicator of the suitability (and potential effectiveness) of the interventions for the target populations, without realizing that a high rate of participation in treatment is a precondition for an internally valid evaluation of the intervention.

### Conclusion

We suggest a straightforward approach to the design and conduct of large trials of psychosocial interventions in populations with an established need for an intervention. First, prior to the design of an RCT, researchers must have a thorough working knowledge of how the intended population relates to the outcome, participation in research, and the intended intervention. Researchers must either use existing, high-fidelity interventions or allow sufficient time to build and pilot test such services prior to starting the project. They should anticipate and minimize potential problems of recruitment, implementation, measurement, participation, and research follow-up. Feasibility, broadly conceived, refers to the viability of all aspects of the proposed intervention and study design and should be determined based on prior research, including pilot studies. Second, any ongoing RCT encountering difficulties that researchers can see will reduce its validity and should be paused or halted. Investigators can then take the time to evaluate the study’s continued viability. Third, when an RCT is completed with compromised validity, researchers should make a good faith effort to expose and explore the problems they encountered by analyzing recruitment, implementation, participation, and attrition. Analyses should be augmented by statistical attempts to reduce, if not eliminate, bias using established methods. Funders should require that researchers designing these studies follow these guidelines. The funding opportunity description should stipulate that study proposals account for these methodological concerns. Funder and investigator adherence to this approach in the design and conduct of large trials would maximize the likelihood that these studies yield useful estimates of intervention effects.
